# Case report of ocular Kaposi’s sarcoma

**DOI:** 10.1186/s12886-017-0525-0

**Published:** 2017-08-14

**Authors:** Jing Yang, Xiao-fang Yin, Yong-ping Li, Shi-you Zhou

**Affiliations:** The State Key Laboratory of Ophthalmology, Zhongshan Ophthalmic Center at Sun Yat-sen University, #54 Xian lie South Road, Guangzhou, 510060 China

**Keywords:** Case report, Ocular Kaposi’s sarcoma, PBSC transplantation, Hhv-8, HIV

## Abstract

**Background:**

Kaposi’s sarcoma (KS) is generally considered a neoplastic disorder of vascular origin and occurs in patients with acquired immunodeficiency syndrome (AIDS) or who have received immunosuppressive treatments after an organ transplant (Soulier et al., Blood 86(4):1276–80, 1995; Viejo-Borbolla and Schulz, AIDS Rev 5(4):222–9, 2003; Schulz, J Antimicrob Chemother 45(Suppl T3):15–27, 2000; Aversa et al. Crit Rev Oncol Hematol 53(3):253–65, 2005; Mbulaiteye and Engels, Int J Cancer 119(11):2685–91, 2006; Tessari et al., Eur J Dermatol 16(5):553–7, 2006). Several Kaposi’s sarcoma case reports involving eyelids and conjunctiva have been published (Bavishi et al., Int J STD AIDS 23(3):221–2, 2012; Baumann et al., Ger J Ophthalmol 4(4):239–45, 1995).

**Case presentation:**

we report a 13 years old asian male patient rare case of ocular KS that was initiated from the sclera and progressed into the cornea and conjunctiva without an human Immunodeificiency Virus (HIV) or HHV-8 infection after a peripheral blood stem cells transplantation. In this case, anti- vascular endothelial growth factor (VEGF) therapy was attempted to stop the advance of ocular lesions and failed. Eventually, the KS was cured by a limbo-corneal lamellar graft, an amniotic membrane and scleral allograft transplantation plus intraoperative mitomycin C(MMC) after the complete excision of the tumors.

**Conclusion:**

A compete surgical excision combined with the intraoperative application of MMC, as well as grafts to repair the scleral, conjunctival, and corneal surfaces, could prevent a recurrence of KS.

## Background

Kaposi sarcoma (KS) is probably a neoplastic disorder of vascular origin and occurs in four forms (classic, endemic, post-transplant and epidemic), which almost have the same histological appearance associated with evidence of human herpes virus type 8 (HHV-8) infection [1–3]. Kaposi’s sarcoma is a common neoplasm in patients with the acquired immunodeficiency syndrome (AIDS) [4]. With the increasing incidence of human immunodeficient virus (HIV) infections, KS have a worldwide distribution now [5]. In the last few years, the tumor has also been frequently diagnosed in patients who receive immunosuppressive treatment after an organ transplant [6, 7]. A number of case reports of KS involving eyelids and conjunctiva in HIV patients have been published [8]. Corneal and scleral location of KS is rare.

We report here a HIV-negative case of extensively involved ocular (cornea, conjunctival and sclera) KS which occurred at 8 months after peripheral blood stem cell (PBSC) transplantation. No recurrence of KS was noted three years after excision of neoplasm combined with lamellar keratoplasty, allogeneic scleral transplantation, intraoperative mitomycin-C application and amniotic membrane transplantation.

## Case report

**Fig. 1:**
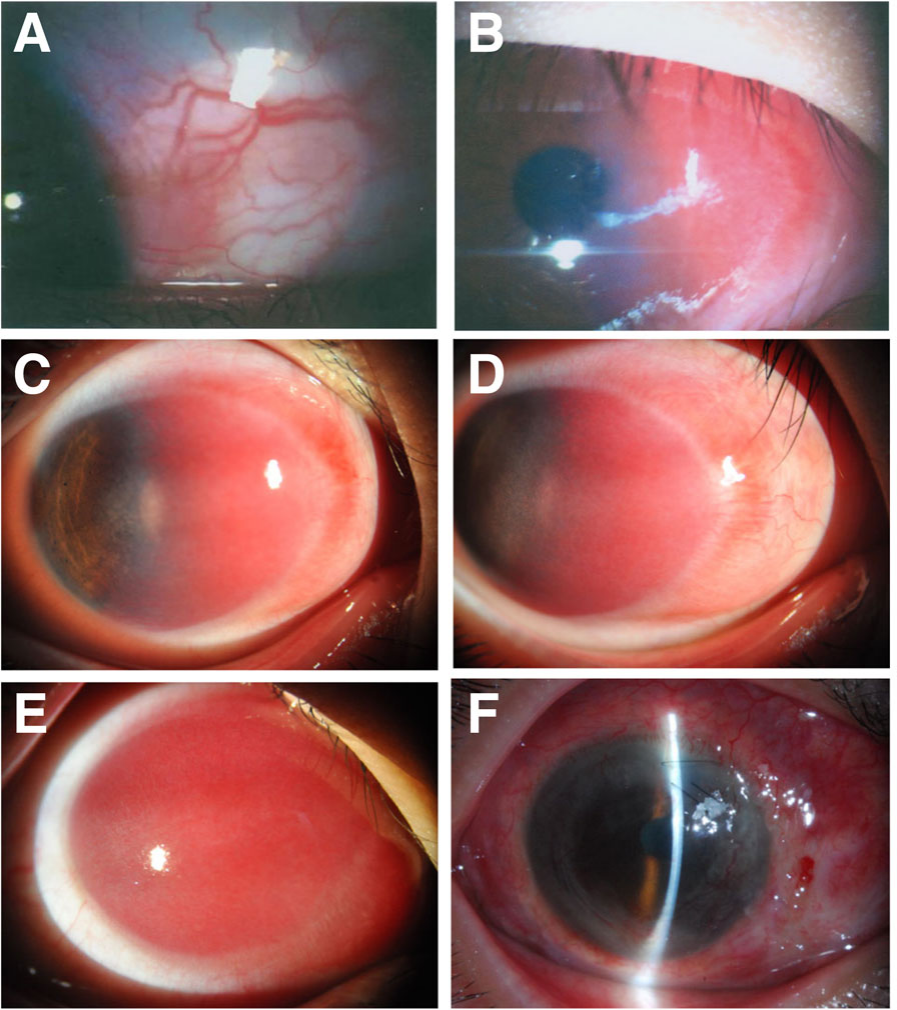
The progress of KS tumors in the eye wall. Panel (**a**) shows the initial ocular presentation. A 4 × 5 mm anciotenic, reddish, slightly elevated (almost invisible) lesion was located on the nasal sclera and separated from the conjunctiva near the cornea limbus of the patient’s right eye. Panel (**b**) shows the solitary lesion had grown larger 6 months later. It grew to nearly 10 × 10 mm, invaded the clear cornea, and almost reached the margo pupillaris. Panel (**c**) shows the red lesion invaded into two thirds of the cornea. The lesion uplifted higher on the sclera and presented a carnification with clear border. Panel (**d**) shows the ocular appearance 2 weeks after a sub-conjunctival injection of bevacizumab. No obvious regression of the neoplasm was observed. The conjunctival congestion was slightly reduced and the nourishing vessels were narrowed. Panel (**e**) shows the completely invaded cornea with a red appearance3months later following the subconjunctival injection. The lesion of the nasal sclera made little progress. A large vessel extended from the temporal sclera to the cornea (arrow). Panel (**f**) shows the ocular surface 1 month after surgery. Many corneal vessels in the peripheral corneal stroma and some conjunctival hemorrhages were noted. No neoplastic mass was observed

A 13-year-old boy presented with a red progressive neoplasm that had been on his right eye for 9 months. The boy had slightly decreased visual acuity without bleeding or obvious irritation before the initial onset (Fig. 1a). Before the appearance of the ocular manifestation, he had undergone an allogeneic peripheral blood stem cells (PBSC) transplantation and subsequent immunosuppressive therapy 17 months earlier as part of the treatment for acute lymphoblastic leukemia. An examination of the bone marrow biopsy showed a complete remission of the acute lymphoblastic leukemia. Furthermore, there were no significant findings in the ocular or social histories.

**Fig. 2:**
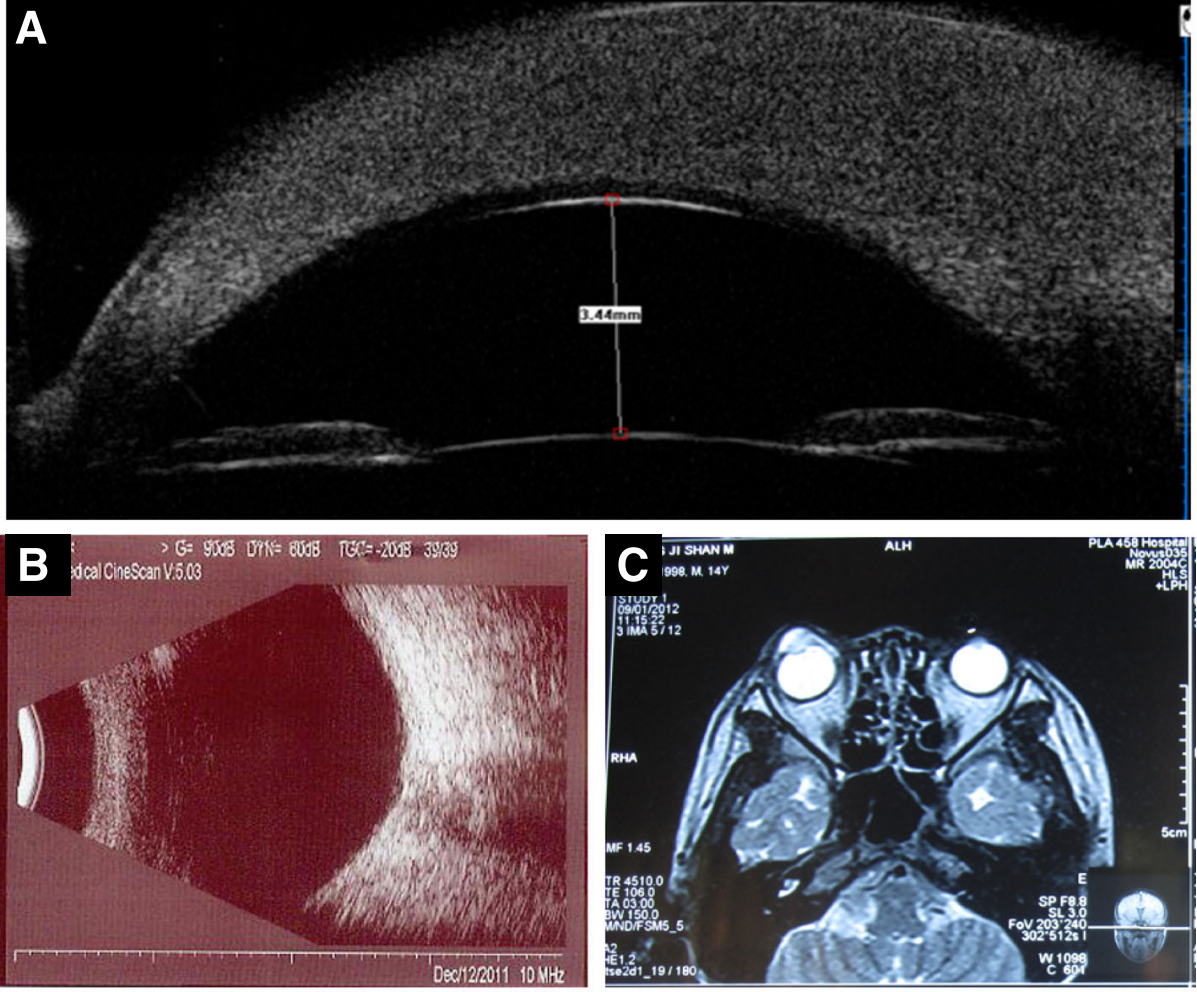
Anterior segment imaging of the diseased eye. Panel (**a**) shows the anterior segment examined by ultrasound bio-microscopy. The whole cornea was invaded by a hypo echoic solid tumor, and almost the full-thickness of the cornea was opacity. Descemet’s membrane can barely be discerned. The central cornea thickness was more than 3000 μm. No obvious abnormalities were found for chamber depth and anterior chamber angle. Panel (**b**) shows ultrasound results of the eye. The ultrasonography shows no remarkable lesions of intraocular structures. However, the color ultrasound shows abundant blood flow signals in the anterior cornea with a velocity of 6 cm/s. Panel (**c**) shows, through magnetic resonance imaging, a space-occupying lesion present in the cornea and conjunctiva. The density was similar to muscle or soft tissue. The eye was integrated and no similar lesions were found in other areas of the head

A telangiectatic, reddish, and slightly elevated lesion of 4 × 5 mm was first noted on the nasal sclera near the corneal limbus of the right eye 8 months after the PBSC transplantation. He was then treated for episcleritis in another hospital with 0.3% tobramycin /0.1% dexamethasone eye drops (TobraDex, Alcon Inc., Texas, USA) four times a day, TobraDex eye ointment every night, and pranoprofen eye drops (Pranopulin, Senju Pharmaceutical Co., LTD, Osaka, Japan) four times daily. When the patient initially came to our clinic, the visual acuity of his right eye became worse. The neoplasm presented as are dish elevated lesion that was 10 × 10 mm in size and had invaded into the cornea and extended close to the margo pupillaris (Fig. 1b). Three months after the patient’s parents refused a biopsy and surgical treatment, the reddish lesion progressed into two thirds of the cornea (Fig. 1c). After being administered a dose of 2.5 mg/0.1 ml subconjunctival bevacizumab (Avastin; Genentech Inc., South San Francisco, CA, USA) per site at the 1 and 5 o’clock positions adjacent to the cornea, the nourishing vessels of the neoplasm became narrowed and the conjunctival congestion was slightly alleviated two weeks later (Fig. 1d). Three months later, the cornea was completely invaded with a red appearance (Fig. 1e) and the lesion progressed slightly at the nasal scleral surface. His visual acuity had decreased to hand motions at a distance of 10 cm. The ultrasound bio-microscopy (UBM) images showed that the whole cornea was occupied by hypo echoic solid tumors and only a thin layer of posterior corneal stroma had normal reflectivity in the center (Fig. 2a). The thickness of the central cornea was more than 3000 μm. The anterior chamber and vitreous cavity of the eye, as well as the cranial bones, were not involved (Fig. 2b, c). The density of reflectivity of the neoplasm that invaded the cornea and conjunctiva was almost the same as that of muscular tissues in magnetic resonance images (Fig. 2c). No similar lesions were found in the other parts of the body. The analysis of whole blood cells and a bone marrow biopsy did not show any evidence of acute lymphoblastic leukemia recurrence, except that the percent of neutrophils decreased to 24.0%, and leukomonocytes and monocytes increased to 64.5% and 8.8%, respectively. Detection of a *Treponema pallidum* infection or viral infection, such as HIV or hepatitis viruses B and C, was also negative.

**Fig. 3:**
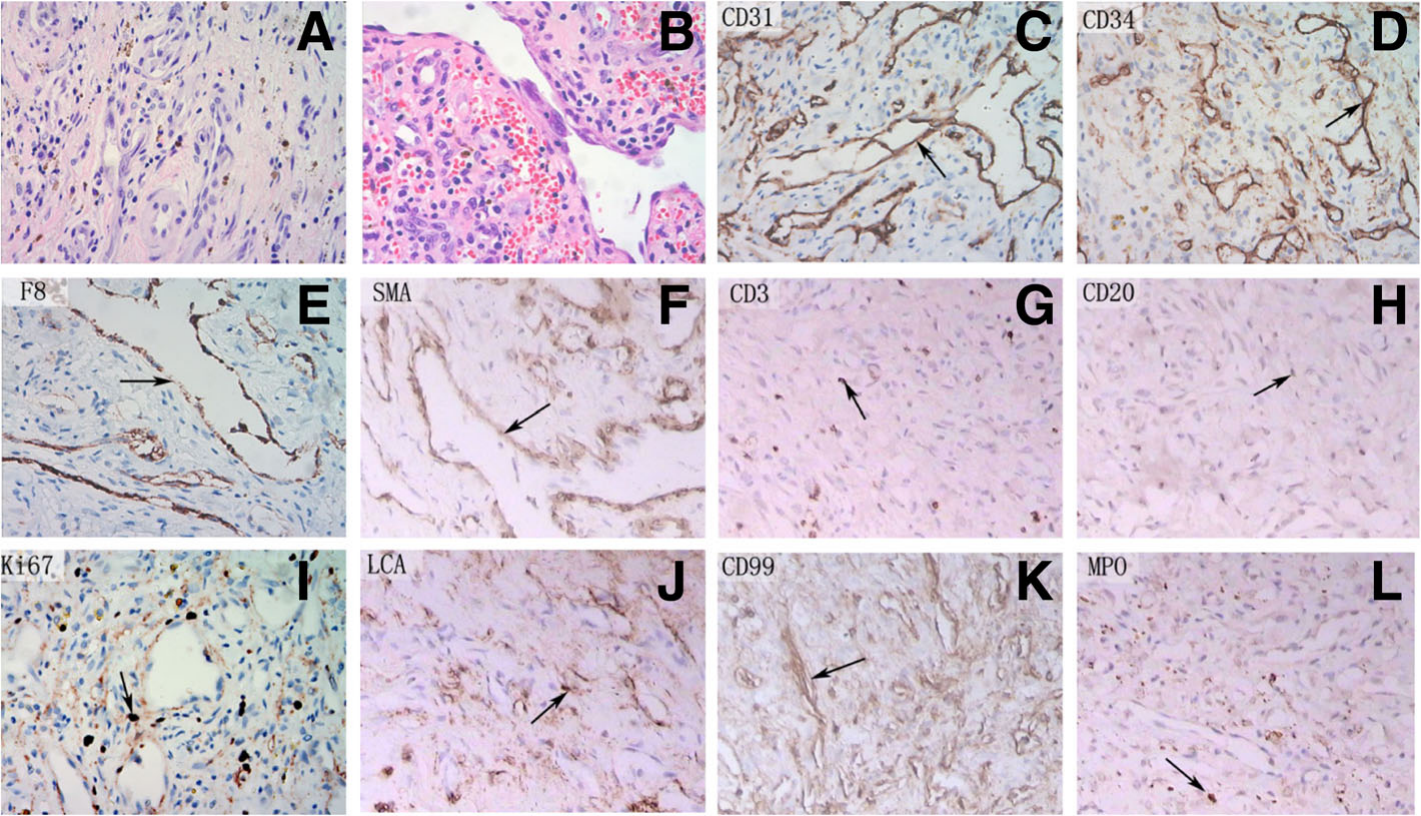
Histopathological and immunohistochemical analyses. Panel (**a**) shows the Kaposi’s sarcoma–like morphology of the tumor. Angiogenic tumors were accompanied with spindle cells and sporadic inflammatory cell infiltrations. A few proliferating spindle cells and irregular cells were distributed inside and outside the neovessels, which presented with a deep dyed large nucleolus, a nuclear membrane coarsening with a distinct nucleolus, pleomorphism, and a few mitotic figures in the nuclei. Panels (**b**–**e**) show the immunohistochemistry results. The vessel walls showed positive staining for CD31, CD34, F8, and smooth muscle actin, which are specific markers for the endothelial cells of blood vessels (**f**, *arrows*). A few lymphocytes were confirmed by positive staining for CD3 and CD20 (**g**, **h**, *arrows*). The lesion was partly positive for Ki67 (about 5%), Leber congenital amaurosis (LCA), and CD99 (**i**–**k**, *arrows*). Individual cells were positive for myeloperoxidase(MPO) (**l**, *arrow*, ×10)

A biopsy of the neoplasm was done with the patient’s and parents’ permissions. Tissues from the involved conjunctiva, sclera, and limbus were excised for histopathological examinations. The hematoxylin-eosin (HE)–stained slides indicated the possibility of angiogenic tumors and a capillary or lymphatic hemangioma. There were a few proliferating spindle cells and other irregular cells both inside and outside the neovessels, which were characterized by deep-dyed large and distinct nucleoli, nuclear membrane coarsening, pleomorphism, and a few mitotic figures in the nuclei (Fig. 3a, b). Immunohistochemical staining with monoclonal antibodies showed positive staining of CD31(1:50 dilution; ab28364; Abcam, Cambridge, UK), CD34 (1:100 dilution; ab8536; Abcam, Cambridge, UK), anti-factor VIII antibody (1:50 dilution; ab139391; Abcam, Cambridge, UK), and anti-alpha smooth muscle actin antibody (1:50 dilution; ab5694; Abcam, Cambridge, UK) in the vessel walls (Fig. 3c–f). A few lymphocyte infiltrations were confirmed by positive staining of CD3 and CD20 (Fig. 3g, h). Some cells were positive for Ki67 (about 5%) (1:100 dilution; ab15580; Abcam, Cambridge, UK), LCA (Leber congenital amaurosis) (1:50 dilution; sc-83,224; Santa Cruz Biotechnology, Guangzhou, China), and CD99 (1:50 dilution; sc-241,354; Santa Cruz Biotechnology, Guangzhou, China) (Fig. 3d). Some cells were also positive for MPO (myeloperoxidase) (1:50 dilution; sc-59,600; Santa Cruz Biotechnology, Guangzhou, China) (Fig. 3i–l). No staining ofHHV-8 (1:50 dilution; ab4103; Abcam, Cambridge, UK) was noted. The DNA amplification of HHV-8 in the excised tissues (conjunctiva, sclera, limbus and cornea) and peripheral blood was also negative. The amplification of HHV-8DNA was performed using the Expand High-Fidelity polymerase chain reaction (PCR) System (Roche Diagnostics, Indianapolis, IN) as previously described [9, 10].

**Fig. 4:**
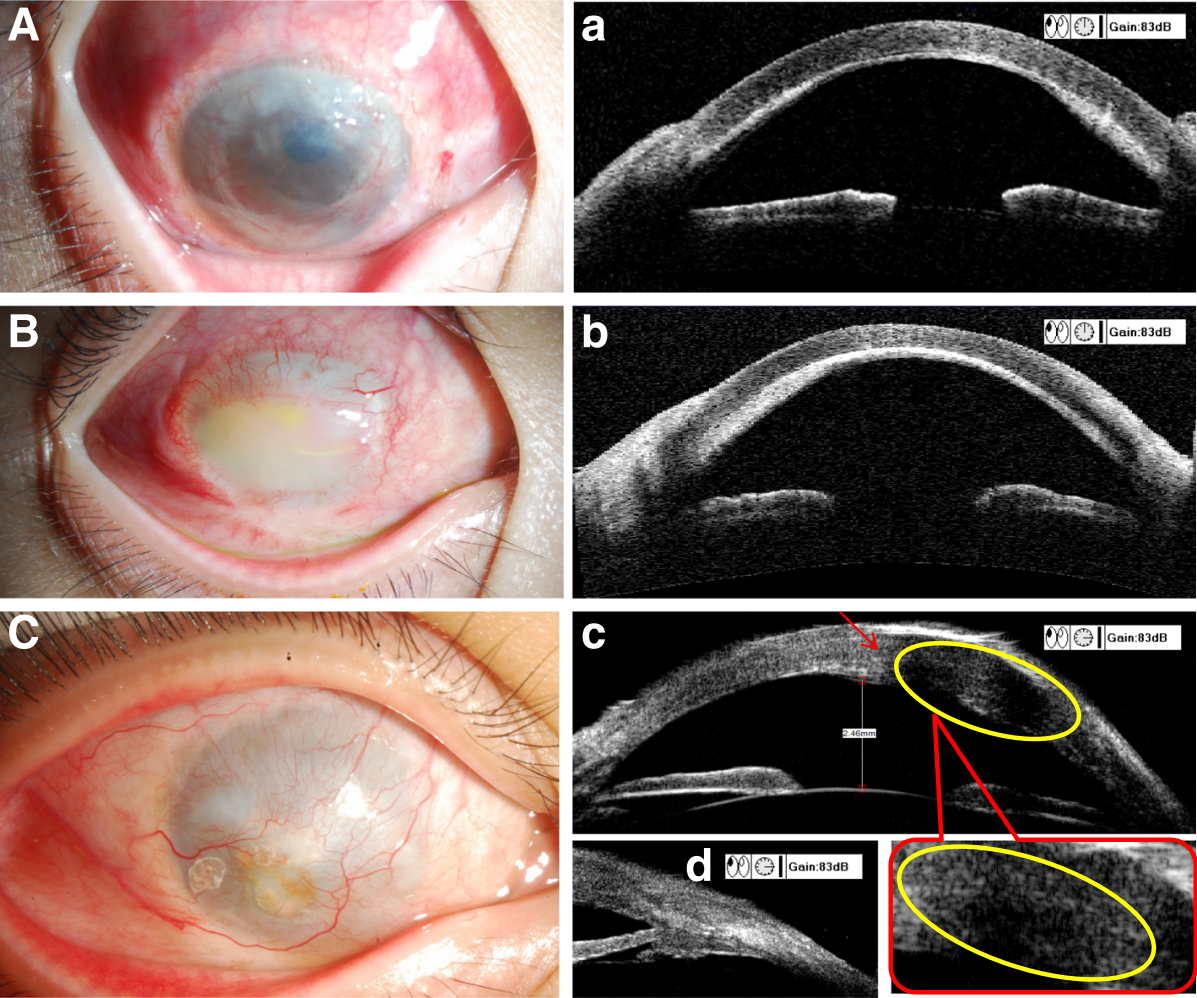
Eye appearance and ultrasound bio-microscopy (UBM) postoperative examination results during the 4-year follow-up period. Panels (**A**) and **a** represent the1-month postoperative period. The lamellar corneal graft was transparent and several corneal neovessels were present in the stroma. There was no corneal thickening. Panels (**B**) and **b** represent the 6-month postoperative period. The corneal graft was opaque and the native corneal stroma was thicker than that in the 1-month postoperative period. Panels (**C**) and **c**–**d** represent the 3 years after the surgery. No corneal thickening and no KS recurrence on the sclera and corneal graft were apparent 3 years after the operation. Panel (**C**) shows complete corneal opacity and neovascularization but no obvious neoplasm recurrence on the cornea, sclera or conjunctiva. Panel **c** shows a slight thickening of the cornea under the corneal graft in the UBM image taken in the past year. Panel **d** shows no obvious thickening of the sclera and conjunctiva in the UBM image taken in the past year

Based on the pathological and clinical manifestations, a diagnosis of KS was established. Surgery was then recommended. During the operation, all of the involved nasal conjunctiva and about 1/2 the thickness of the involved sclera was removed. When the corneal lesions were dissected, only a small amount of posterior corneal stroma remained transparent under the surgical microscope. Before repair of the ocular surface defects, a pre-immersed cotton swab with 0.2 mg/ml MMC was placed onto the exposed residual sclera and medial rectus muscle for 5 min. Then, a full thickness limbo-corneal lamellar corneal graft without Descemet’s membrane and endothelium was transplanted onto the corneal surface. An alcohol-preserved full-thickness heterogeneous sclera was tailored and sutured to fit the scleral defect. Finally, the conjunctival defect was repaired with an amniotic membrane graft (Fig. 1f). One month after surgery, the lamellar graft was transparent and a great deal of corneal neovascularization presented in the peripheral corneal stroma (Fig. 4A) with a normal anterior chamber depth and without corneal thickening (Fig. 4A). From 6 months after surgery to the subsequent observation 3 years later, the corneal graft became opaque and neovascularization occurred (Fig. 4B, C) without corneal bugling (Fig. 4B). No recurrent KS was observed on the sclera or corneal graft. Neither the conjunctival surface nor the cornea have had hyperplasia or a malignant growth tumor over the last 3 years (Fig. 4C). However, in the past year, the UBM examination observed a slight thickening of the native corneal stroma under the lamellar cornea graft (Fig. 4C) without a sclera or conjunctiva neoplasm (Fig. 4D).

## Discussion

This was a rare case of ocular KS in which the conjunctiva, cornea, and sclera were extensively involved after a PBSC transplantation without an HIV infection.

KS was first described in 1872. It has four clinical forms: classic, endemic, epidemic, and a post-transplant form [11]. The classic form is usually seen on the lower extremities as violaceous macules and plaques. The endemic form of KS was recognized in Central Africa and described long before the HIV pandemic [12–14]. The epidemic form is also referred to as the HIV–related KS and is the most aggressive form. The post-transplant form of KS has been frequently diagnosed in patients with treatment of exogenous immunosuppression, typically after a solid organ transplantation. The reduction or discontinuation of immunosuppression can often cause complete remission of KS [15]. The KS–associated herpes virus (KSHV; also known as HHV-8) is the infectious agent that causes this neoplasm. The HHV-8 infection is necessary for KS to develop, but it is not sufficient for the complete development of KS and cofactors exist. In this study, the patient had a history of PBSC transplantation and did not have lesions in areas other than the ocular surface, and the ocular lesions did not go into remission after the discontinuation of immunosuppressive treatments. Additionally, no viral infections, including HIV and HHV-8, were detected in this patient’s ocular tissues and blood.

There are several reports of KS after solid organ transplantations, especially after renal transplantations [16–18]. However, the incidence of KS following bone marrow (BM) or PBSC transplantations is uncommon. The intensive chemotherapy used during the preparation for a transplant, the use of irradiation-based conditioning therapy, and the severe immunosuppression after transplantation may play key roles in the development of secondary neoplasms [19, 20]. However, there is no clear association between a dose or duration of immunosuppressive therapy and the development of iatrogenic KS [21]. The patient in this study only had oral cyclosporin A (CsA; 150 mg q12h) for 3 months and the blood concentration of CsA was maintained in a safe range.

Viral infections are considered to be a contributory factor that may activate neoplasm formation [22]. Recently, some studies have found DNA sequences of HHV-8 (or KS–associated herpes virus) in KS tissue samples [23, 24]. Infection of HHV-8 is thought to precede the development of KS and be one of the factors leading to bone marrow failure after transplantation [25]. However, no HHV-8 DNA sequences were detected in the dissected tissues and blood by PCR in this study.

Reports of eye wall involvement in KS are rare. Conjunctival or eyelid KS usually grows slowly and can often be cured with a simple excision. Radiotherapy, chemotherapy, amniotic membrane transplantation, and intraoperative application of MMC have also been introduced mostly as adjunctive interventions combined with a local excision to treat KS [26–28]. In this case, anti-VEGF therapy was attempted to stop the advance of ocular lesions and failed. Eventually, the KS was cured by a limbo-corneal lamellar graft and an amniotic membrane and scleral allograft transplantation plus intraoperative MMC after the complete excision of the tumors. However, the native corneal stroma was slightly thickened and the neovasularized lamellar corneal graft was not bugling. No recurrent neoplasm on the sclera and conjunctiva have been observed by UBM examinations in the past year. It is difficult to determine if the thickening of the posterior native corneal stoma is hyperplasia, inflammation, or a KS recurrence. Thus, a long-term follow-up period is needed to be maintained.

## Conclusions

In summary, we have reported a rare case of ocular KS that was initiated from the sclera and progressed into the cornea and conjunctiva without an HIV or HHV-8 infection after a PBSC transplantation. A compete surgical excision combined with the intraoperative application of MMC, as well as grafts to repair the scleral, conjunctival, and corneal surfaces, could prevent a recurrence of KS. There were no risks of distant metastasis during the years of follow-up examinations.

## Abbreviations

AIDS: Acquired immunodeficiency syndrome
BM: Bone marrow
CsA: Cyclosporin A
HE: Hematoxylin-eosin
KS: Kaposi’s sarcoma
KSHV also known as HHV-8: KS–associated herpes virus
LCA: Leber congenital amaurosis
MMC: Mitomycin C
MPO: Myeloperoxidase
PBSC: Peripheral blood stem cells
PCR: Polymerase chain reaction
UBM: Ultrasound bio-microscopy
VEGF: Vascular endothelial growth factor
